# Rotationplasty for Unplanned Fixation of Pathological Fracture Distal Femoral Osteosarcoma

**DOI:** 10.1155/2020/8813619

**Published:** 2020-10-27

**Authors:** Chindanai Hongsaprabhas, Wittavat Chenboonthai, Phoomchai Suvaraksakul, Chris Charoenlap

**Affiliations:** ^1^Department of Orthopaedics, Faculty of Medicine, Chulalongkorn University and King Chulalongkorn Memorial Hospital, Thai Red Cross Society, 1873 Rama IV Road, Pathumwan, Bangkok 10330, Thailand; ^2^Department of Orthopaedics, Wetchakarunrasm Hospital, 38/2 Liap Wari Road, Krathum Rai, Nong Chok, Bangkok 10530, Thailand; ^3^Department of Orthopaedics, Surin Hospital, 68 Lanckmeong Road, Nai Meong, Meong District, Surin 32000, Thailand; ^4^Department of Orthopaedics, Faculty of Medicine, Chulalongkorn University, 1873 Rama IV Road, Pathumwan, Bangkok 10330, Thailand

## Abstract

**Introduction:**

Rotationplasty had been reported as a salvage procedure for many decades. However, this procedure has not been used for unplanned fixation for pathological fracture of osteosarcoma. Therefore, this is the first case report of rotationplasty for this particular indication. *Case Presentation*. We report a case of a 22-year-old Thai female patient who sustained a supracondylar fracture at the distal femur and underwent a surgical treatment by open reduction and internal fixation with a distal femoral locking plate and screws. Follow-up radiographic imaging revealed that there were abnormal osteolytic lesions, and conventional high-grade osteosarcoma was diagnosed by a pathological study. There were no distant metastases from Computed Tomography (CT) scan or Technitium-99m bone scintography. After discussing with the patient for treatment options, rotationplasty was chosen for her definitive treatment after 3 courses of neoadjuvant chemotherapy. All of the contaminated tissues were removed during the surgery. The neurovascular bundles were preserved. A standard rotationplasty type A-1 according to the Winkelmann Classification was performed. Postoperative imaging showed satisfactory outcomes, and the wound healed uneventfully. The patient was able to move her ankle as a knee, and external prosthetic fitting was made. Adjuvant chemotherapy was given after a free margin with good tumor necrosis which was achieved as shown in the pathological study. At the patient's 3-year follow-up visit, she has stable size of lung nodules. She can walk with external prosthesis, limping slightly. Her new knee could move as expected, and she was satisfied with the result of the treatment.

**Conclusion:**

Rotationplasty for unplanned fixation of pathological fracture is a complex procedure. Patients often do not select this type of treatment because of the cosmetic acceptance even though it yields a good functional result. Therefore, awareness of the pathological fracture should initially be taken into account to prevent inappropriate fixation which could result in an unnecessary amputation.

## 1. Introduction

Pathological fracture of osteosarcoma has an adverse prognostic factor for patient survival [1]. Limb sparing surgery may be achieved if the tumor has minimally contaminated the surrounding tissue and there is a good response to the chemotherapy [1–3]. However, when an unplanned fixation was undergone and the degree of contamination was unacceptable, a limb sparing surgery would not be a good option [4, 5]. Nevertheless, rotationplasty could be offered to the patient instead of high amputation above the knee, and the outcome for this operation was good. Moreover, rotationplasty had never been reported as the salvage procedure for this indication.

## 2. Case Presentation

**Figure 1:**
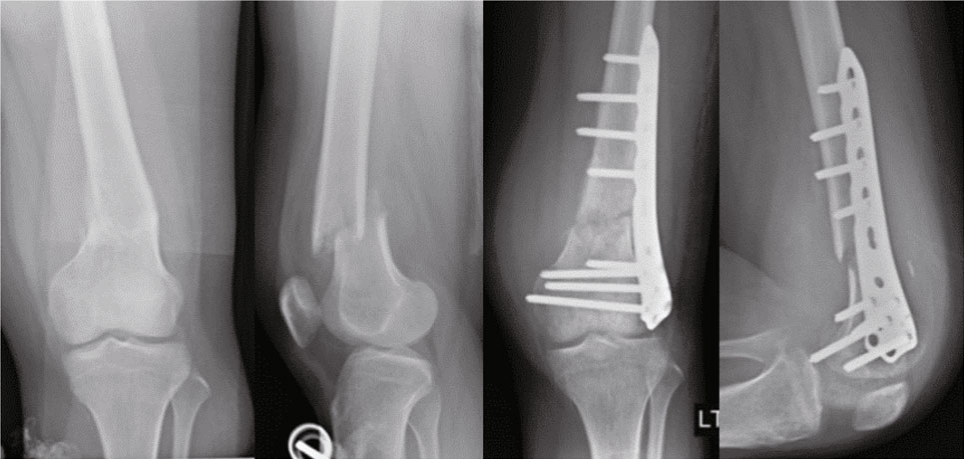
Pre- and postoperative X-ray of the unplanned fixation of the pathological fracture femur.

**Figure 2:**
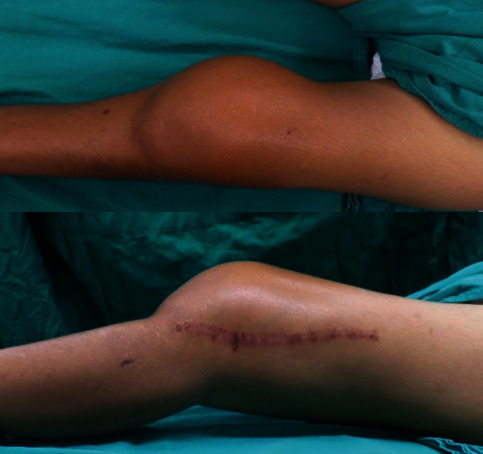
Three months after bony fixation, a bone mass was developed.

**Figure 3:**
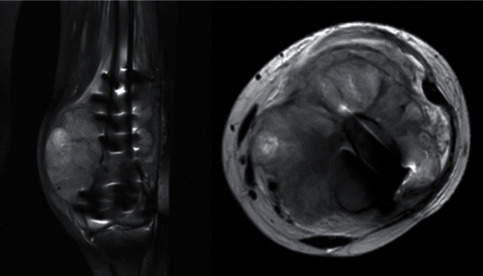
MRI scans of the bone mass that developed 3 months after operation.

We report a case of a 22-year-old female patient with a history of left leg pain for 1 month when she fell and sustained a supracondylar fracture at the distal femur. She underwent a surgical treatment by open reduction and internal fixation with a distal femoral locking plate and screws. Follow-up radiographic imaging revealed proper bony and implant alignments (Figure 1). However, abnormal osteolytic lesions were retrospectively detected at the fracture site. Few months later, she experienced a progressive enlarged mass prominent at the medial aspect of the left knee with severe night pain that brought her to visit her physician again (Figure 2). A magnetic resonance imaging (MRI) scan was performed, and a cortical bone destructive lesion with soft tissue mass at the distal femur was detected as hypointense signal intensity (SI) on T1-weighted, hyperintense SI on T2-weighted, and STIR sequence along with radiating heterogenous enhancement (Figure 3). The patient was then referred to King Chulalongkorn Memorial Hospital with a diagnosis of a progressive enlarging tumor at the left distal femur. An incisional biopsy was done, and the result indicated that the patient had conventional high-grade osteosarcoma. There were no distant metastases from the Computed Tomography (CT) scan of the chest or Technitium-99m bone scintography. Three courses of cisplatin and doxorubicin were given to her as the neoadjuvant chemotherapy, and the MRI was repeated. The results were satisfactory.

**Figure 4:**
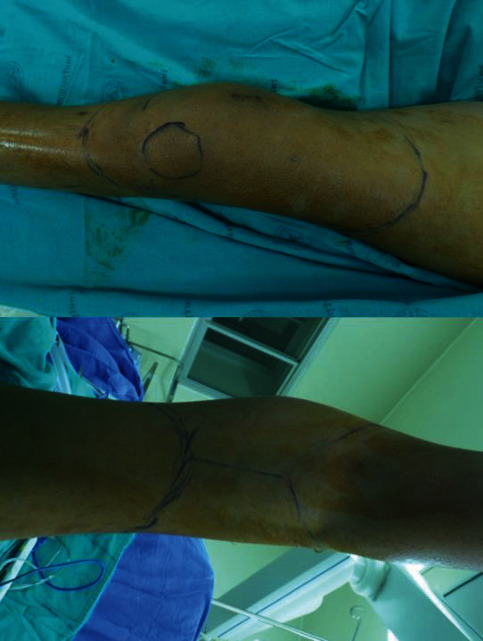
Intraoperative steps of the surgery began by outlining the areas that will be incised.

**Figure 5:**
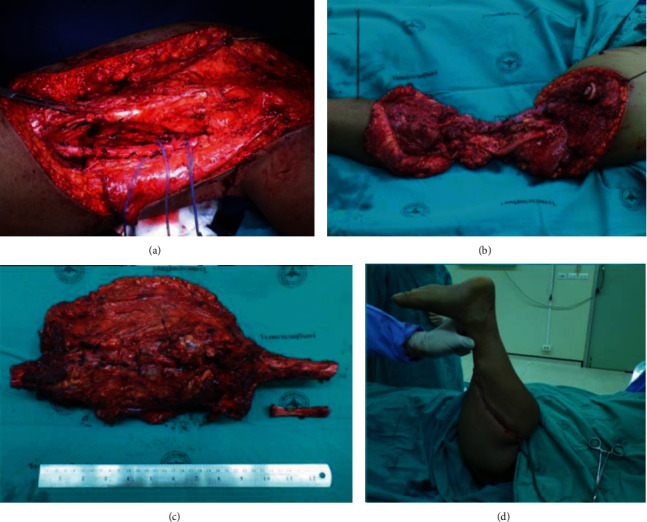
Popliteal artery was identified and protected (a). Tumor was removed leaving behind the continuity of uncontaminated neurovascular bundle (b). The specimen was sent for pathological study (c). The distal leg was rotated and reattached to the proximal thigh (d).

**Figure 6:**
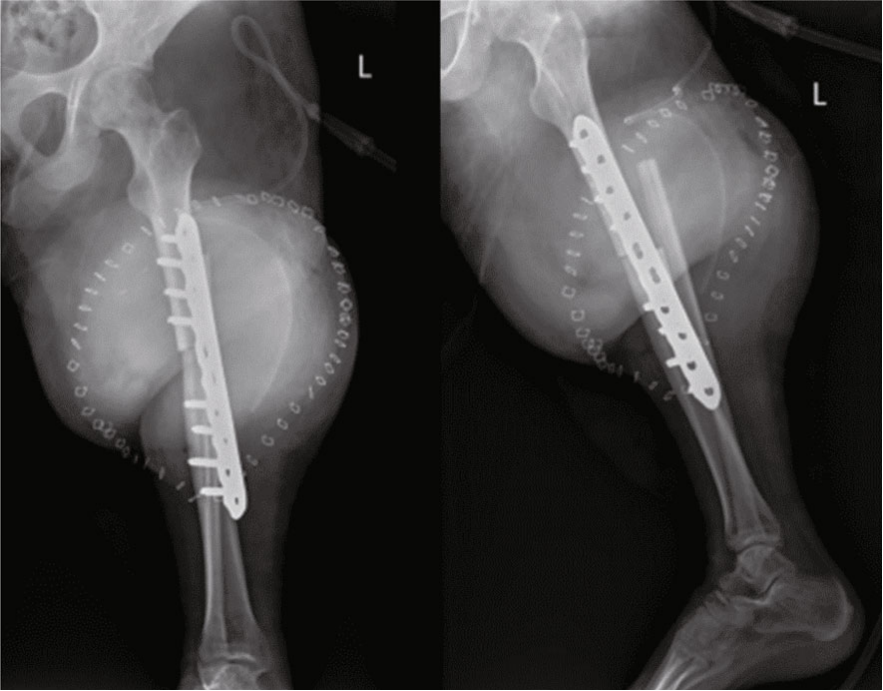
Postoperative X-rays of the surgical site.

**Figure 7:**
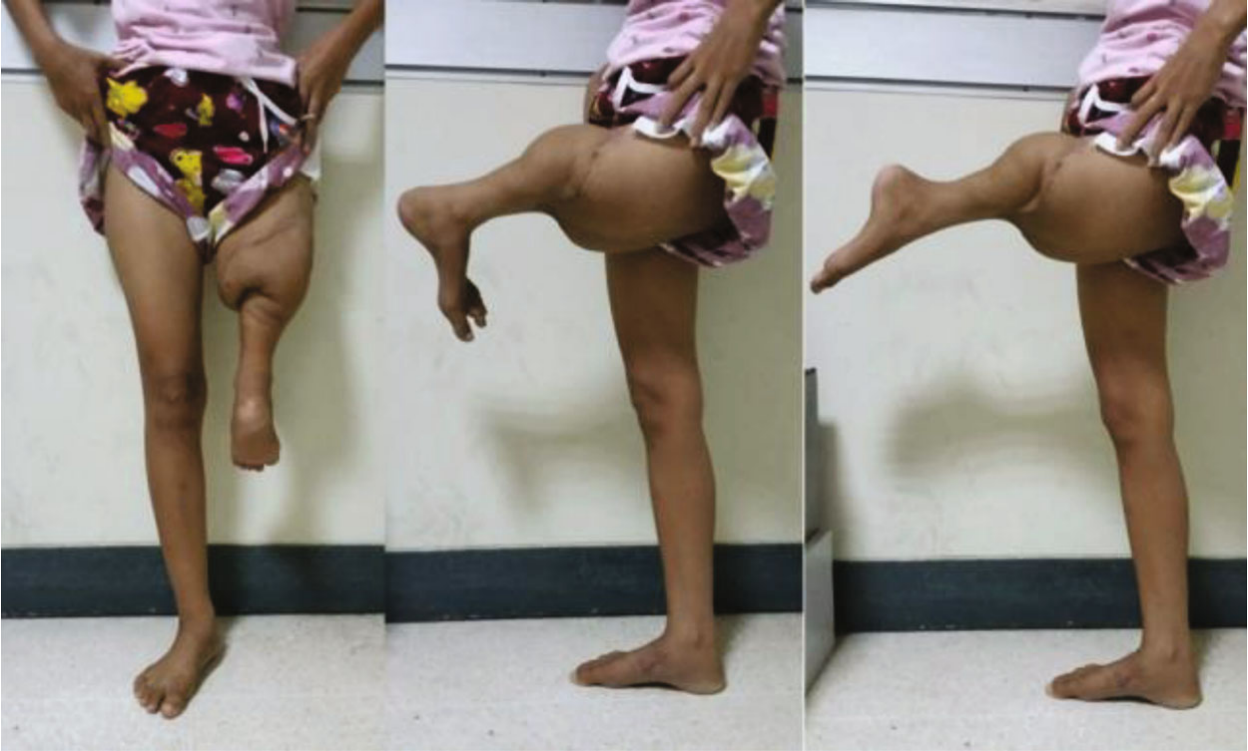
Clinical functional outcome at the 3-month follow-up visit; she could move her new knee without limitation.

Treatment options were discussed with the patient. The patient opted for the standard rotationplasty type A-1 according to the Winkelmann Classification [6] as her definitive treatment. The operation was scheduled at Surin Hospital which was nearby her hometown. The plan of resection was made based on the repeated MRI after neoadjuvant chemotherapy. The skin incision was designed into a rhomboid shape with the apex at the level of the proximal bone cut and the distal arms meeting at the level of the distal cut. The two oblong arms of the skin incision met posteriorly both proximally and distally. The posterior cuts were then joined by a single vertical posterior incision (Figure 4). Femoral vessels, sciatic nerve, and common peroneal nerve were identified and protected (Figure 5(a)). Circumferential soft tissue along with the tumor was en bloc resected aiming for an adequate margin. Temporary Kirschner wire fixations were inserted above the femur and below the tibia to propose the resection level as guidance for the orientation (Figures 5(b) and 5(c)). The distal limb was externally rotated to 180 degrees and reattached to the femur while preventing vascular kinging during the turn (Figure 5(d)). The locking compression plate and screws were fixed on the posterolateral femur and anteromedial tibia, and soft tissue was sutured layer by layer. Postoperative imaging yielded satisfactory results, and the wound healed uneventfully (Figure 6). The patient was able to move her ankle as a knee and external prosthetic fitting was made. Adjuvant chemotherapy was given after a free margin with good tumor necrosis which was achieved from pathological study. At her 3-year follow-up visit, she is alive and doing well. She has a stable size of lung nodules. She can walk with the external prosthesis, limping slightly. Her new knee could move as expected, and she was satisfied with the result of the treatment (Figure 7). Furthermore, she just recently gave birth.

## 3. Discussion

Pathological fracture was considered one of the adverse prognostic factors for survival and outcome of treatment for osteosarcoma patients [1]. However, limb sparing surgery is still possible without compromising the overall survival rate [1–3]. Unplanned fixation of this condition can result in worse outcome and survival because the surrounding soft tissue area was contaminated with the tumor [4]. Most of the cases with a distal femoral lesion needed to be amputated high above the knee or hip disarticulation [5].

Rotationplasty had been reported as a salvage procedure for many conditions [7–9]. The principle of this operation was to preserve the knee function after removing the bone around the knee from any causes by converting the ankle joint to replace the position. The indications were mostly for a very young child because there is the potential of bone remodeling. The major drawback was the cosmetic acceptance from the patients and caregivers [7, 10–12].

Historically, Salzer et al. [12] first reported the use of rotationplasty for sarcomas of the lower extremity; then, it was popularized by Kotz and Salzer in 1982 [11]. Then, Winkelmann classified rotationplasty into two main types, A and B, which were further divided into many subtypes [6]. In this patient, rotationplasty type A was performed while preserving major neurovascular bundles. However, if the margin of control became vulnerable with contamination of the vessels, a vascular bypass surgery may be necessary, and vascular anastomosis did not increase the risk of rotationplasty failure [13]. Many incision and surgical techniques had been reported to facilitate the ease of the operation while minimizing the complications [10]. However, a large tumor and preoperative pathological fracture may increase the rate of failure [14].

Prosthetic fitting in patients who underwent rotationplasty is one of the critical points to resume a high level of function and return to premorbid physical activities [15]. The prosthetic knee joint should use a double-action orthotic ankle joint to compensate for the patient's inability to control the knee flexion moment at the heel strike, thus providing knee stability. A long-term follow-up of children who underwent rotationplasty revealed that the smaller discrepancies between the residual thigh-shank relative to the contralateral thigh resulted in a better gait and functional score in adulthoods [16]. Even though the main indication for rotationplasty is mostly for young children, we believe that in carefully selected adults, this particular operation may offer many benefits to the patient as seen with our patient who did not have tumor recurrence at 3 years post surgery.

## 4. Conclusion

This is the first case report of rotationplasty for the indication of unplanned fixation of osteosarcoma with pathological fracture. It is a complex procedure and often not selected as a choice of treatment. However, this procedure has a good functional result. Awareness of the pathological fracture should initially be taken into account to prevent inappropriate fixation which could result in an unnecessary amputation.

## Data Availability

No data were used to support this study.
